# 小鼠Lewis肺癌原位模型的构建

**DOI:** 10.3779/j.issn.1009-3419.2010.01.08

**Published:** 2010-01-20

**Authors:** 馨 刘, 治平 伍, 曙光 左, 永春 周, 艳 陈, 熙才 王

**Affiliations:** 650118 昆明，昆明医学院第三附属医院（云南省肿瘤医）肿瘤研究所 Cancer Institute of the Tird Hospital Afliated Kunming Medical College, Kunming 650118, China

**Keywords:** 肺肿瘤, 动物模型, 小鼠, Lung neoplasms, Animal models, Mice

## Abstract

**背景与目的:**

肺癌原位模型包括小鼠自发性肺肿瘤模型和气道内接种模型等，但自发性肺肿瘤模型耗时较长且成瘤率不能保证，而气道内接种模型成瘤部位及大小不稳定。本研究以3LL细胞系细胞接种于C57BL/6小鼠肺原位，与皮下接种模型比较，探讨其稳定性、转移特性，并建立小鼠肺原位癌模型的优化方法。

**方法:**

将不同数量级的3LL细胞分别直接接种于C57BL/6小鼠左侧腋下制备皮下接种模型和以Matrigel悬浮后接种于其左肺制备原位接种模型，观察两种模型的生存期，并对小鼠进行解剖后行病理切片检查、免疫组化染色检测微血管密度、流式细胞仪检测CD44v。

**结果:**

皮下组成瘤率分别为100%、66.7%、16.7%，未见明显转移。原位组成瘤率分别为100%、100%、83.3%，并可转移至对侧胸廓及肺脏。原位组中位生存期（38 d、35 d、23 d）明显少于皮下接种组（82 d、72 d、50 d）。原位接种组微血管密度（120.2±9.73）高于皮下组（92.6±7.12）。原位接种组肿瘤细胞悬液CD44v表达（26.46±1.56）%高于皮下接种组（23.13±1.02）%。

**结论:**

以3LL细胞接种于小鼠肺部所建立的肺癌原位模型简单可靠，重复性高，具有较皮下接种模型更强的转移特性。

## Abstract (en)

**Background and objective:**

The mouse lung cancer orthotopic model includes spontaneous lung cancer model and endotracheal transplanted model, and etc. The spontaneous lung cancer needs longer time and does not ensure the rate of the generation of the tumor; as for endotracheal transplanted model, the position and size of the tumor are instable. In this study, the 3LL cell line was orthotopically transplanted into the lung of the C57BL/6 mice, compare to the heterotopic model, to discuss their stability and transfer-characteristics. And this study was also to optimize the method of establishing lung cancer orthotopic animal model.

**Methods:**

Different quantity of 3LL cells were inoculated into the left oxter of C57BL/6 mice to establish the heterotopic model; or suspended with Matrigel then inoculated into the left lung of C57BL/6 mice to establish orthotopic model. The survival-time of the mice was examined. The tissue was collected for the subsequent histology assay after euthanizing the mice. Microvessels density (MVD) was observed and counted by immunohistological chemistry. CD44v was detected by flow cytometry.

**Results:**

TTumor-form-rate of the heterotopic group were 100%, 66.7%, 16.7%, respectively, and had no macroscopic transfer. Tumor-form-rate of the orthotopic group were 100%, 100%, 83.3%, respectively, and had widespread transfer in contralateral chest and the lung. The median survival time of the orthotopic group (38, 35, 23 days) were less than the heterotopic group (82, 72, 50 days). MVD of the orthotopic group (120.2±9.73) was higher than the heterotopic group (92.6±7.12). The expression of CD44v of orthotopic (26.46± 1.56)% was higher than the heterotopic group (23.13±1.02)%.

**Conclusion:**

The lung cancer orthotopic model which established by 3LL cells transplanted into the lung of the mice is simple, dependable, repeatable and has stronger transfer characteristics than the heterotopic model.

动物模型的建立是研究非小细胞肺癌生物学特性和各种体内治疗实验研究的基础。该模型必须具备可重复性，并具备最为近似的肿瘤微环境及机体环境。Sheel等^[1]^以苯并芘对A/J小鼠灌胃构建肺自发肿瘤模型，28周后解剖，仅有80%左右的成瘤率。国内用类似方法诱发大鼠肿瘤，16周后才出现肿瘤，且肿瘤类型不一，包括鳞癌、腺癌等^[2]^。Zou等^[3]^用细针将人肺癌细胞系H358和H460送至裸鼠的气管内构建肺癌原位移植模型，但肿瘤生长部位、数目及大小都不稳定。为建立简易、稳定、可重复性好的肺癌原位模型，本研究以3LL细胞系细胞直接接种于C57BL/6小鼠肺内，并与皮下接种模型比较，对其稳定性、转移特性及对小鼠肺原位癌模型构建方法的优化进行探讨。

## 1 材料与方法

### 1.1 3LL细胞的培养及生长曲线的绘制

3LL细胞株购自上海市胸科医院胸部肿瘤研究所。用含10%胎牛血清的RPMI-1640完全培养基，加双抗（100 U/mL青霉素，100 μg/mL链霉素），置于37 ℃、5%CO_2_混合气体孵箱中培养，每1天-2天换液，每3天传代一次，传代用0.25%胰酶消化。取1×10^4^细胞接种于24孔板，每24 h计数，重复3孔，持续8天，计算每孔细胞均数，绘制生长曲线。

### 1.2 细胞悬液的制备

取对数生长期的3LL细胞，以0.25%的胰酶消化，收集细胞，离心去上清，用无菌生理盐水洗涤两次，将细胞悬浮于生理盐水中，台盼蓝染色细胞活力测定大于90%，并进行细胞计数，调整细胞浓度分别为2×10^5^/mL、2×10^6^/mL、2×10^7^/mL，立即对小鼠进行皮下接种及肺原位接种。

### 1.3 实验动物和接种方法

实验动物C57BL/6小鼠72只，雌雄各半，6周龄，购自北京维通利华实验动物技术有限公司[许可证号：syxk（京）2006-0024]。

24只小鼠采用皮下接种法，具体如下：①分组：将小鼠随机分为3组，每组8只，其中2只于20 d时处死，行免疫组化检测及流式检测，另外6只用于生存曲线的监测。第1组接种细胞总数为1×10^4^，第2组接种细胞总数为1×10^5^，第3组接种细胞总数为1×10^6^。②接种：对小鼠左侧腋下皮肤进行酒精消毒，以1 mL注射器将50 μL细胞悬液注入小鼠左侧腋下，稍停针后拔针。

48只小鼠采用原位接种法，具体如下：①分组：将小鼠随机分为3组，每组16只，其中10只用于定时监测肿瘤形成情况，另外6只用于生存曲线的监测。第1组接种细胞总数为1×10^4^，第2组接种细胞总数为1×10^5^，第3组接种细胞总数为1×10^6^。②麻醉：戊巴比妥钠溶液50 mg/kg腹腔内注射，将戊巴比妥钠配成10 mg/mL的工作液，按0.05 mL/10 g体重剂量对小鼠进行麻醉。③接种：小鼠麻醉后，将其仰卧固定在操作台上，对小鼠前胸壁进行酒精消毒，在左腋前线肋弓上约1.5 cm处作一约5 mm的小切口，分离皮肤及皮下组织暴露至胸壁，将50 μL细胞悬液与50 μL Matrigel混匀，在快凝固之前以胰岛素注射针将3LL细胞与Matrigel混悬液共100 μL注入小鼠左肺，进针约3 mm，注射完后停针5 s，拔针后缝合切口。

### 1.4 移植瘤生长的观察和测量

定期观察C57BL/6小鼠的精神、饮食、排便、体重和活动等情况，皮下接种组于肿瘤形成后第3天开始测量肿瘤大小，以游标卡尺测量肿瘤长径及短径，以公式V=a^2^bπ/2（a为肿瘤的短径，b为肿瘤的长径）计算肿瘤大小。原位移植组分别在接种后第3、6、9、12、20天，处死2只小鼠，观察肿瘤形成及转移情况，并于第20天行免疫组化及流式检测。

### 1.5 病理学检测

留取小鼠肿瘤、肺脏、肝脏行病检。病理HE染色按常规制备病理切片，二甲苯脱蜡2次，各10 min，无水乙醇洗去二甲苯2次，各1 min；95%酒精1 min，90%酒精1 min，85%酒精1 min，自来水冲洗2 min；苏木精染色4 min，自来水冲洗1 min；1%盐酸酒精分化20 s，自来水冲洗1 min；伊红染色1 min，自来水洗30 s；70%酒精脱水20 s，90%酒精脱水30 s，95%酒精脱水2次，各1 min，无水乙醇2次，各2 min；二甲苯3次，各2 min；中性树胶封片，显微镜下鉴定小鼠肺脏肿瘤灶。

### 1.6 免疫组化检测及微血管密度（microvessels density, MVD）计算

免疫组化以石蜡切片按DAB染色试剂盒说明操作，一抗为抗CD31抗体。按照参考文献中的方法计算微血管密度^[4]^。每张切片在低倍镜（×100）下确定肿瘤内5个血管密集区，再在高倍镜（×200）下计数每个密集区中1个视野的微血管数，以5个区域微血管数的平均数作为微血管密度（MVD），在高倍镜（×200）下拍照。

### 1.7 流式检测

解剖小鼠分离肿瘤组织，研磨过滤收集单细胞悬液，以磷酸盐缓冲液（phosphate buffer solution, PBS）洗涤后调整细胞浓度为1×10^6^/mL，加入一抗（CD44v, Abcam）孵育0.5 h后，PBS洗涤，加二抗孵育0.5 h。PBS洗涤后上机检测。

### 1.8 统计学分析

结果采用SPSS 16.0统计学软件进行*Kaplan-Meier*生存分析及方差分析，比较皮下组及原位接种组生存曲线、肿瘤微血管密度及CD44v的差异，*P* < 0.05为差异有统计学意义。

## 2 结果

### 2.1 3LL细胞生长曲线

体外培养的3LL细胞在倒置显微镜下呈梭形（图 1），持续8天的细胞计数观察发现3LL细胞于接种第3天逐渐进入快速增殖期，第6天后增殖速度变缓（图 2）。

**1:**
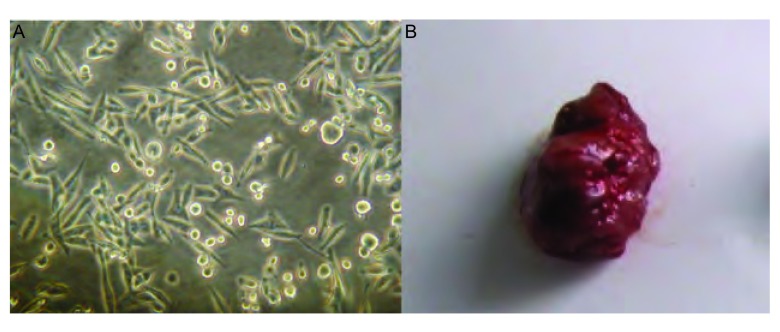
3LL细胞和皮下移植瘤 3LL cell and heterotopic lung cancer

**2:**
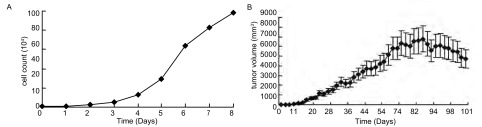
生长曲线 Growth curves

### 2.2 移植瘤的形成及动物的生存情况

皮下接种1×10^6^剂量级的小鼠最先成瘤，术后第7天即可于大部分小鼠左侧腋下触及米粒大小肿块，成瘤率100%；1×10^5^剂量级小鼠于14天后成瘤，成瘤率66.7%；1×10^4^剂量级小鼠20 d后成瘤，但成瘤率仅16.7%。小鼠恶液质出现较晚，生存期较长，最长可达3个月，3组中位生存时间分别为82 d、72 d、50 d。后期肿瘤生长巨大，最大可达（6 769.44±815.35）mm^3^，并出现坏死（图 1、图 2）。

原位接种组1×10^6^剂量级的小鼠最先成瘤，在术后3天解剖的2只小鼠中即有1只出现肺部肉眼可见的瘤结节，从第9天以后的所有老鼠均成瘤，12天出现对侧肺的转移，2周-3周后小鼠严重消瘦，行动困难，恶液质状态；1×10^5^剂量级的小鼠在第9天解剖时有成瘤迹象；1×10^4^剂量级的小鼠至12天开始才有成瘤迹象，成瘤率均为100%（图 3）。第3组小鼠于第22天时就开始出现死亡，其中位生存期为23 d，第2组小鼠中位生存期为35 d，第1组为38 d，存活最长者为50 d（图 4）。生存分析显示原位组、皮下组组内及组间比较均有统计学差异（*P* < 0.05）。

**3:**
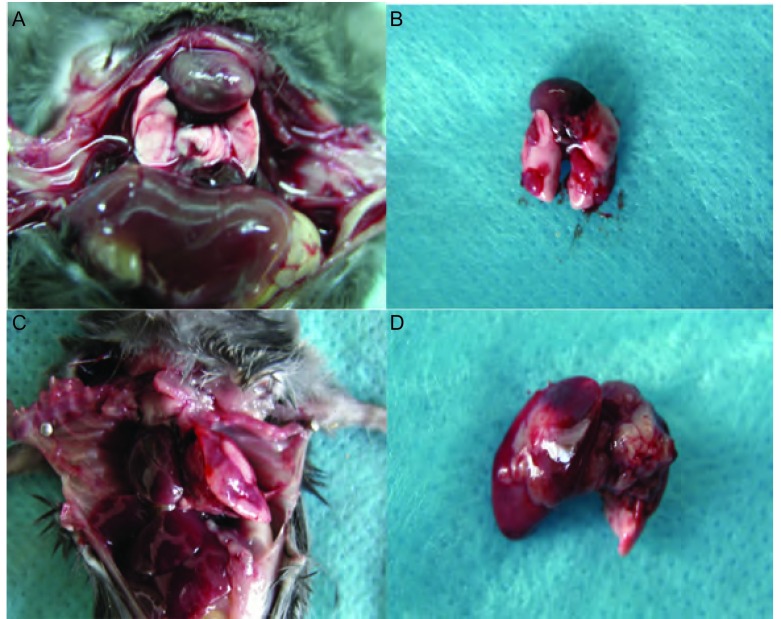
小鼠肺原位癌模型 The orthotopic lung cancer model in mice

**4:**
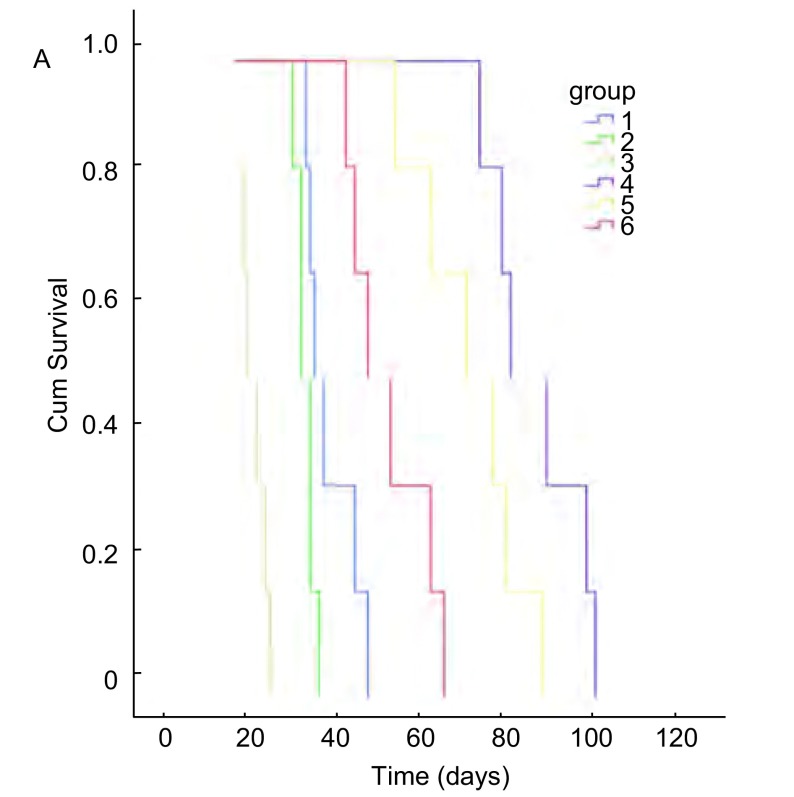
各组小鼠的生存曲线 Survival curves of the mice in different groups

### 2.3 病理检查

皮下接种组肿瘤组织局部浸润，侵及皮肤及肋骨，但未见远处器官转移。原位接种组对小鼠肺组织进行病理切片检查，可见肺正常组织间聚集有片状核大深染的肿瘤细胞。CD31免疫组化检测微血管密度，接种第20天原位接种组（120.2±9.73）高于皮下组（92.6±7.12）（图 5，图 6）。

**5:**
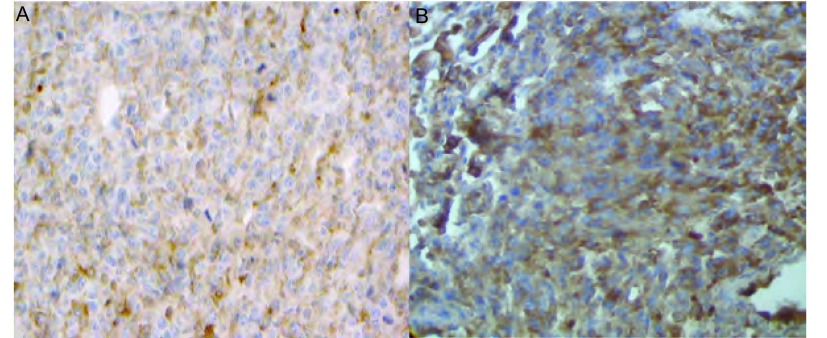
CD31表达情况（免疫组化DAB染色，×200） CD31 expression in different group (immunohitochemical DAB staining, ×200)

**6:**
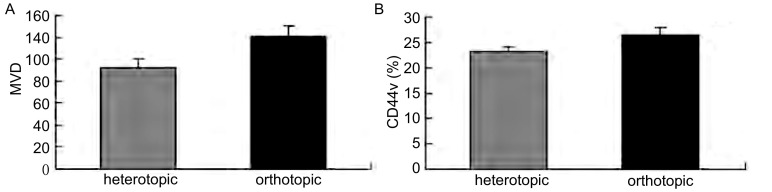
MVD计数和CD44v的表达 MVD count and the expression of CD44v

### 2.4 流式检测

两组肿瘤细胞均有CD44v的表达，原位接种组肿瘤细胞悬液（26.46±1.56）%表达高于皮下接种组（23.13±1.02）%（图 6）。

## 3 讨论

大约80%的肺癌为非小细胞肺癌，化疗和放疗对非小细胞肺癌都缺乏有效的治疗，因此探索肺癌的发病机制及新的肺癌治疗方法意义重大，而进行相关研究的基础就在于肺癌模型的构建。

小鼠肺肿瘤模型包括小鼠自发性肺肿瘤模型及移植瘤模型。其中，小鼠自发性肺肿瘤模型与人肺癌具有相似的形态学、组织学及分子生物学特性^[5]^，但其主要问题是所需要的成瘤时间过长，成瘤率不能保证。

相对来说，移植瘤模型因其制作简单、成瘤率稳定及所需成瘤时间较短被各实验室广泛运用。Pagets的种子和土壤学说^[6]^证实了肿瘤细胞的表型受到器官微环境的影响，皮下移植瘤模型转移发生率较小，而且生存曲线的数据不准确。因此，肿瘤的原位移植动物模型是目前较为理想而可靠的肿瘤模型。原位接种的方式又包括了支气管内注射和肺内注射两种方法，支气管注射成瘤及瘤体大小、数目均不稳定。有实验室将A549细胞注入裸鼠肺内，构建移植人肺癌细胞的小鼠原位肺癌模型^[7]^，但在这一模型中裸鼠B细胞系统缺陷使小鼠体内免疫反应不能完全模拟人的免疫反应，同时裸鼠的低免疫力容易导致裸鼠感染死亡，而在有免疫泄露的情况下，则可能导致成瘤率降低，这一切均可导致实验误差。况且裸鼠价格昂贵，进行大规模的试验性实验往往造成不必要的浪费。所以在新的治疗方法的研究中，仅少数研究者采用了人肺癌的原位模型。

3LL细胞是小鼠Lewis肺癌，来源于C57小鼠，本研究选用3LL细胞对C57小鼠进行接种，比较皮下接种及原位接种两种造模方式。本研究发现若采用皮下接种方式，低剂量组成瘤率低（16.7%），成瘤时间长，约需2周-3周；高剂量组成瘤率虽能达到100%，但存在小鼠生存时间不一、转移不稳定的问题。而在原位接种组中，仅低剂量组1只小鼠未能成瘤，其原因考虑为接种时细胞有渗漏，导致细胞数未达1×10^4^，其余两组小鼠成瘤率均为100%，大部分小鼠均有对侧肺的转移。通过血管转移是肺癌的主要转移方式之一，为证实原位接种组肿瘤具有较强的转移能力，本研究首先对两组的微血管密度进行了比较，发现肿瘤接种后第20天，原位接种组的微血管密度高于皮下接种组，这可能与原位接种部位血供充足，肿瘤组织生长迅速有关。其次，本研究对肿瘤细胞表面的CD44v分子进行了检测。CD44是表达于内皮细胞细胞膜的单链糖蛋白，在IL-5、TNF-α、EGF等细胞因子活化下，通过HER-2、c-Src、Tiam 1以及RhoA等信号途径^[8]^，可导致细胞迁移及不可控性的细胞增殖。CD44v是CD44的变异体，在肿瘤细胞的侵袭性生长及转移中起着重要的作用。有研究^[9]^表明CD44v介导产生的粘附基质对于肿瘤细胞转移后的定居及生长至关重要，CD44v诱导的抗凋亡信号通路的活化支持转移的形成，而且CD44v还可以将CD44v相关的MMP-9、MMP-2蛋白聚集在细胞膜，剪切TGF-β抑制相关蛋白，活化抑制的TGF-β，从而诱导血管形成^[10]^。因此，CD44v的表达与肺癌的转移相关^[11]^。本研究发现肿瘤接种20 d后原位接种肿瘤CD44v的表达水平高于皮下接种组，这一结果具有两方面的意义：一方面部分解释了原位接种肿瘤具有较强转移特性的可能原因；另一方面，这可能是原位接种组微血管密度较高的原因之一。当然CD44v的表达受到多种细胞因子的调节^[8]^，在原位接种环境中表达上调的因素有待进一步的研究。

在造模过程中，我们对原位接种法做了优化，依照文献使用了Matrigel，并对注射方法进行了调整。Matrigel的特性使接种的肿瘤细胞能够固定在注射部位，并成为肿瘤细胞生长的支架，使肿瘤形成单一病灶，便于观察肿瘤的转移。而注射以盐水混悬的肿瘤细胞后，肿瘤细胞会因为重力作用而广泛分散，导致肿瘤的多发病灶^[12]^。在我们的研究中，并不是所有小鼠均形成了单一病灶，预实验中有的小鼠两侧肺脏布满肿瘤结节，并与胸膜广泛粘连，其原因在于Matrigel的凝固时间较长，如果将Matrigel与肿瘤细胞混匀后直接注射，Matrigel进入肺脏之后不能立即凝固，必定产生肿瘤细胞在肺内的扩散，随后我们改进了方法，将Matrigel与肿瘤细胞混匀后，室温孵育数分钟，待混悬液显得粘稠之后再将其注入小鼠肺脏，即可形成单一肿瘤病灶。然后，肿瘤在接种肺进行性生长并且发生了相邻部位、胸淋巴结及对侧肺的转移。肺癌的这种胸内转移模式与肺癌临床患者的相似，表明这种模型具有很强的临床相关性。但是，在这种模型中，缺乏临床所显现的远处转移，这可能是因为肺部及胸廓的广泛转移导致了小鼠呼吸功能严重衰竭，在还未来得及出现远处转移的时候，小鼠因全身衰竭而死^[13]^。

综上所述，以悬于Matrigel的3LL细胞对C57小鼠肺部原位接种以制备小鼠肺原位癌模型，较皮下接种模型具有更好的成瘤率及转移特性。
